# Prenatal Exposure to Endocrine-Disrupting Chemicals and Subsequent Brain Structure Changes Revealed by Voxel-Based Morphometry and Generalized Q-Sampling MRI

**DOI:** 10.3390/ijerph18094798

**Published:** 2021-04-30

**Authors:** Chao-Yu Shen, Jun-Cheng Weng, Jeng-Dau Tsai, Pen-Hua Su, Ming-Chih Chou, Shu-Li Wang

**Affiliations:** 1 Institute of Medicine and School of Medicine, Chung Shan Medical University, Taichung 40201, Taiwan; shenchaoyu@gmail.com (C.-Y.S.); fernand.tsai@msa.hinet.net (J.-D.T.); jen@csh.org.tw (P.-H.S.); cshy1146@csh.org.tw (M.-C.C.); 2 Department of Medical Imaging, Chung Shan Medical University Hospital, Taichung 40201, Taiwan; 3 Bachelor Program in Artificial Intelligence, Department of Medical Imaging and Radiological Sciences, Chang Gung University, Taoyuan 33302, Taiwan; 4 Medical Imaging Research Center, Institute for Radiological Research, Chang Gung University and Chang Gung Memorial Hospital at Linkou, Taoyuan 33302, Taiwan; 5 Department of Psychiatry, Chang Gung Memorial Hospital, Chiayi 613, Taiwan; 6 Department of Pediatrics, Chung Shan Medical University Hospital, Taichung 40201, Taiwan; 7 Division of Thoracic Surgery, Department of Surgery, Chung Shan Medical University Hospital, Taichung 40201, Taiwan; 8 National Institute of Environmental Health Sciences, National Health Research Institutes, Miaoli 350, Taiwan

**Keywords:** phthalate esters, perfluorochemicals (PFCs), heavy metals, voxel-based morphometry (VBM), generalized q-sampling imaging (GQI)

## Abstract

Previous studies have indicated that prenatal exposure to endocrine-disrupting chemicals (EDCs) can cause adverse neuropsychiatric disorders in children and adolescents. This study aimed to determine the association between the concentrations of prenatal EDCs and brain structure changes in teenagers by using MRI. We recruited 49 mother–child pairs during the third trimester of pregnancy, and collected and examined the concentration of EDCs—including phthalate esters, perfluorochemicals (PFCs), and heavy metals (lead, arsenic, cadmium, and mercury)—in maternal urine and/or serum. MRI voxel-based morphometry (VBM) and generalized q-sampling imaging (GQI) mapping—including generalized fractional anisotropy (GFA), normalized quantitative anisotropy (NQA), and the isotropic value of the orientation distribution function (ISO)—were obtained in teenagers 13–16 years of age in order to find the association between maternal EDC concentrations and possible brain structure alterations in the teenagers’ brains. We found that there are several specific vulnerable brain areas/structures associated with prenatal exposure to EDCs, including decreased focal brain volume, primarily in the frontal lobe; high frontoparietal lobe, temporooccipital lobe and cerebellum; and white matter structural alterations, which showed a negative association with GFA/NQA and a positive association with ISO, primarily in the corpus callosum, external and internal capsules, corona radiata, superior fronto-occipital fasciculus, and superior longitudinal fasciculus. Prenatal exposure to EDCs may be associated with specific brain structure alterations in teenagers.

## 1. Introduction

Environmental endocrine disrupting chemicals (EDCs) include phthalate esters, perfluorochemicals (PFCs), heavy metals, etc. They are groups of chemicals that are widely used, such as in industrial applications and consumer products. EDCs can be absorbed from the air, water, food, and earth into the human body and disrupt the endocrine system and regulation of hormones.

Phthalate esters include monobutyl phthalate (MBP), monobenzyl phthalate (MBzP), and metabolites of di-(2-ethylhexyl) phthalate (DEHP), including mono-2-ethylhexyl phthalate (MEHP), mono-2-ethyl-5-oxohexyl phthalate (MEOHP), and mono-2-ethyl-5-hydroxohexyl phthalate (MEHHP). They are commonly used in plasticizer products, for example, in food packaging, clothing, building materials, and children’s toys. They can enter the human body via inhalation and ingestion, and may cause endocrine disruption that may affect growth and fertility. A previous animal study mentioned that prenatal phthalate exposure may influence rat brain development [1]. Some human studies have revealed that exposure to phthalate esters may cause social problems and a tendency to be reckless and impulsive, and may even lead to attention-deficit/hyperactivity disorder (ADHD) symptoms [2,3,4]. Furthermore, several studies have noted that exposure to phthalate esters may induce abnormal frontal function and decreased intelligence quotient (IQ), which may degrade learning and cognitive abilities [5,6]. Therefore, the prevalent use of phthalate esters has become a public health issue, which may affect brain development and children’s behaviors [3].

PFCs include perfluorooctane sulfonate (PFOS), perfluorooctanoic acid (PFOA), perfluorododecanoic acid (PFDoA), perfluorononanoic acid (PFNA), and perfluoroundecanoic acid (PFUA). PFCs have been produced for more than a half-century, and are widely used in modern commercial and industrial products to aid in processing polymers, surface coatings, pharmaceuticals, and surfactants in cleaning products. PFCs are highly resistant to chemical, thermal, and biological degradation, and can migrate from the products and enter the environment [7]. Animal and human exposure occurs mainly through polluted food, water, and air/dust intake. Exposure to PFCs, especially during early life, could be harmful, and could result in a range of adverse health effects, including disrupted immune and neuroendocrine systems, as well as hepatic toxicity and neurotoxicity [8]. Some studies have mentioned that PFOA exposure was associated with kidney, pancreas, liver, and testicular cancer [9,10]. One study showed that serum concentrations of PFNA correlated with serum-free T4 levels in adolescents and young adults [11]. Another study mentioned that PFCs may affect fetal brain development and increase the risk of congenital cerebral palsy, especially in the male sex [12].

Regarding the heavy metals, a previous marine mammal study mentioned that the highest concentrations of manganese (Mn), iron (Fe), zinc (Zn), selenium (Se), and mercury (Hg) were noted in the liver, whereas cadmium (Cd) predominantly accumulated in the kidneys, and that those concentrations of heavy metals might cause severe neurological damage [13]. One study mentioned that widespread exposure to lead (Pb) continues to cause neurological deficits and diseases [14]. In the 1960s, Pb was emerging as a major public health problem that might cause permanent brain damage [15]. Furthermore, several studies have reported that heavy metals can transfer across the placenta to the fetus, and may cross the blood–brain barrier (BBB) to directly affect the central nervous system, which may subsequently contribute to adverse neurodevelopmental outcomes involving cognition and sensorimotor functions [16,17,18,19].

Although multiple neurobehavioral effects of EDC exposure have been observed in animal and human studies, most of the studies have looked at relatively limited types and numbers of EDCs. In fact, the general population could be simultaneously exposed to multiple EDCs, and the potential effects of other substances that might be present were often ignored. In addition, although evidence from brain imaging studies examining EDC effects in humans is sparse, a recent study used high-resolution T1-weighted MRI images to evaluate brain-structural anomalies in children exposed prenatally to a widely used organophosphate pesticide—chlorpyrifos (CPF)—and the results showed significant cerebral abnormalities associated with higher prenatal CPF exposure, including in the frontal, temporal, parietal, and occipital lobes [20]. Therefore, in this study, we attempted to explore the relationships between brain volume/structure and prenatal exposure to several common EDCs, including phthalate esters (MBP, MBzP, and 2 important metabolites—MEHP and MEOHP), PFCs (PFOS, PFOA, PFDoA, PFNA, and PFUA), and heavy metals (Pb, Cd, Hg, and inorganic arsenics). Prenatal exposure was determined via maternal blood and urine collected during the third trimester of pregnancy, and children at the age of 13–16 years old underwent brain MRI with voxel-based morphometry (VBM) to evaluate brain volume changes, and generalized q-sampling imaging (GQI), which was based on the diffusion method with unique q-space reconstruction, to investigate brain microstructure and the integrity of anatomical connectivity [21,22,23]. To the best of our understanding, this is the first study to focus on human brain MRI structural observations in relation to prenatal exposure to multiple EDCs

## 2. Materials and Methods

### 2.1. Participants and EDCs Measurements

In the study, we recruited 49 mother–child pairs from the general population (26 male and 23 female children) in central Taiwan from previous cohort studies [3,24]. The recruited mothers were 25–34 years old, and all of them delivered at a designated medical center and without complications—such as eclampsia or pre-eclampsia—during the pregnancy and delivery periods. The prenatal EDCs in urine and/or serum were collected, and should be sufficient for analysis of the results. The recruited teenagers had no neurologic or psychiatric disorders, and they cooperated well during the MRI scans. We examined the association between phthalate esters and heavy metals in the maternal urine and PFCs in the maternal blood collected during the third trimester of pregnancy, and the teenagers’ brain MRIs at 13 to 16 years of age (mean = 13.9, standard deviation = 0.47) using 3 Tesla MRI machines in the Chung Shan Medical University Hospital.

The examined phthalate esters’ metabolites included MBP, MBzP, MEHP, MEOHP, and DEHP (MEHP + MEOHP + MEHHP). The examined PFCs included PFOS, PFOA, PFDoA, PFNA, and PFUA. The examined heavy metals included Pb (*n* = 47), Cd (*n* = 47), As (*n* = 45), and Hg (*n* = 21 for maternal serum; 43 for umbilical cord blood). Most of the studied EDCs were analyzed at the National Institute of Environmental Health Sciences, National Health Research Institutes (NIEHS/NHRI), Taiwan, but Hg and PFCs were analyzed at National Taiwan University. The limits of detection (LOD) for the EDC concentrations, and the percentages of study participants with EDC concentrations below their LOD, were recorded. The EDC concentrations under the LOD were replaced as half the LOD value for analysis. The details of the EDC analysis were mentioned in our previous studies [3,24]. All subjects gave their informed consent for inclusion before they participated in the study. The study was conducted in accordance with the Declaration of Helsinki, and the protocol was approved by the Ethics Committee of Chung Shan Medical University Hospital (CS15069).

### 2.2. MRI Data Acquisition

All participants underwent a brain MRI examination on a 3T imaging system (Skyra, Siemens, Germany) with a 20-channel head/neck coil. A three-dimensional magnetization-prepared rapid gradient-echo imaging (3D MPRAGE) sequence was used to obtain T1-weighted images for VBM analysis. The images were acquired with the following parameters: repetition time (TR) = 2500 ms; echo time (TE) = 2.27 ms; inversion time (TI) = 902 ms; flip angle = 8°; voxel size (resolution) = 1 × 1 × 1 mm^3^; total slices = 160; and scan time = 5.8 min. Diffusion images for GQI analyses were obtained with TR/TE = 4800/97 ms; FOV = 250 × 250 mm^2^; matrix = 128 × 128; slices = 35; in-plane resolution = 2 × 2 mm^2^; slice thickness = 4 mm; signal average = 1; 64 × 3 noncollinear diffusion weighting gradient directions, with b = 1000, 1500, 2000 s/mm^2^; and 12 null images without diffusion weighting (b = 0 s/mm^2^). The scan time was approximately 16.5 min.

### 2.3. The Association between Phthalate Esters, PFCs, Heavy Metals, and VBM

All structural data were processed using Statistical Parametric Mapping 8 (SPM8, Wellcome Department of Cognitive Neurology, London, UK) with the Voxel-Based Morphometry 8 (VBM8, University of Jena, Department of Psychiatry, Jena, Germany) toolbox. The raw data were first normalized to the International Consortium for Brain Mapping (ICBM)’s East Asian Brain templates. Subsequently, whole-brain T1-weighted images were segmented into gray matter and white matter, and were normalized to adjust for differences in volume. Finally, all segmented images were smoothed with a Gaussian kernel to increase the signal-to-noise ratio.

After image preprocessing, multiple regression with false discovery rate (FDR) correction was used to obtain the association between the maternal urine concentrations of prenatal phthalate ester metabolites (MBP, MBzP, MEHP, MEOHP, and DEHP), maternal blood PFC concentrations (PFOS, PFOA, PFDoA, PFNA, and PFUA), and maternal urine/blood concentrations of heavy metals (Pb, Cd, As, and Hg), and the gray and white matter volumes of the teenagers’ brains. Gender, IQ, family income, and whole-brain volume were used as covariates, and adjusted for when appropriate to evaluate the independent effects of EDC exposure. For the creatinine correction, creatinine was used as a covariate. In addition, in the heavy metals studied, we used maternal creatinine-corrected urine concentrations as another creatinine correction method. Standard T1WIs were implemented using SPM8 as the underlying map. A *p*-value of less than 0.05 was considered statistically significant and represented by the T-score, which was given by SPM and calculated using a t-distribution with *n* − 2 degrees of freedom. The formula for the test statistic is t = *r* √(*n* − 2)/√(1 − *r*^2^). 

### 2.4. The Association between Phthalate Esters, PFCs, Heavy Metals, and GQI Indices

For the GQI analysis, initially, eddy current correction was performed using FSL (FMRIB, Oxford, UK), followed by registration of the diffusion images with the b0 (null) image in native diffusion space using linear transformation. Finally, the registered images were mapped to the standard T2 template after affine transformation, with 12 degrees of freedom and nonlinear warps, using SPM8. After the preprocessing procedure, GQI index mapping—including generalized fractional anisotropy (GFA), normalized quantitative anisotropy (NQA), and the isotropic value of the orientation distribution function (ISO)—were reconstructed from multi-shell diffusion data using DSI Studio (National Taiwan University, Taipei, Taiwan) [21]. For the statistical analysis, multiple regression analysis with FDR correction was used to obtain the association between the maternal urine concentrations of prenatal phthalate ester metabolites (MBP, MBzP, MEHP, MEOHP, and DEHP), maternal blood PFC concentrations (PFOS, PFOA, PFDoA, PFNA, and PFUA), and maternal urine/blood concentrations of heavy metals (Pb, Cd, As, and Hg), and the brain GQI indices in the teenagers. Gender, IQ, and family income were used as covariates. For the creatinine correction, creatinine was used as a covariate. In addition, in the heavy metals studied, we used maternal creatinine-corrected urine concentrations as another creatinine correction method. GQI indices were used as the underlying map. A *p*-value of less than 0.05 was considered statistically significant and represented by T-score, which was given by SPM and calculated using a t-distribution with *n* − 2 degrees of freedom. The formula for the test statistic is t = *r* √(*n* − 2)/√(1 − *r*^2^). 

The results of the brain structures significantly associated with EDCs in the GQI analysis further underwent Pearson’s partial correlation analysis for confirmation using Statistical Analysis System Enterprise Guide 6.1 (SRS EG 6.1, Institute Inc., Cary, NC, USA). Natural log-transformed values of EDC metabolites were used as independent variables. For creatinine correction, creatinine was used as a covariate. In addition, in the heavy metals studied, we used maternal creatinine-corrected urine concentrations as another creatinine correction method. Potential outliers of all the variables were deleted to fit normal distribution. The mean values of the ROI of the significantly associated brain structures were used as dependent variables. Gender, IQ, and family income were used as covariates. A value *r* of between 0 and 0.3 (0 and −0.3) was defined as a weak positive (negative) correlation; between 0.3 and 0.7 (−0.3 and −0.7) was defined as a moderate positive (negative) correlation; and between 0.7 and 1 (−0.7 and −1) was defined as a strong positive (negative) correlation. A *p*-value of less than 0.05 was considered statistically significant. 

## 3. Results

### 3.1. Demographic Characteristics

Table 1 summarized the EDC concentrations in urine/serum samples from pregnant women. As had the highest median concentration among the heavy metals. MBP had the highest median concentration among the phthalate esters (except for ΣDEHP, which represents a number of phthalate metabolites). PFOS had the highest median concentration among the PFCs. From the concentration results, we observed that the interquartile range of each EDC was large. This indicates that the EDC levels were differentially distributed among pregnant women in this study, which may have been due to different lifestyles, diets, environmental exposures, etc (Table 1).

The LOD for urinary MBP, MBzP, MEHP, MEHHP, and MEOHP were 1.6, 0.99, 0.55, 0.26, and 0.23 μg/L, respectively, and the corresponding proportions below the LOD were 0%, 6.1%, 0%, 10%, and 2%, respectively. The LOD for serous PFOS, PFOA, PFDoA, PFNA, and PFUA were 0.11, 0.45, 0.07, 0.10, and 0.13, respectively, and the corresponding proportions below the LOD were 0%, 21.3%, 23.4%, 6.4%. and 6.4%, respectively. The LOD for urinary Pb and Cd were 0.022 and 0.066 μg/L, respectively, and no sample had urinary Pb or Cd levels below the LOD. The As exposure was the sum of 4 As species—arsenite, arsenate, monomethylarsonate (MMA), and dimethylarsinate (DMA)—representing total inorganic As exposure. The LOD were 0.09, 0.05, 0.05, and 0.04 μg/L for arsenite, arsenate, MMA, and DMA, respectively, and the corresponding proportions below the LOD were 29.4%, 45.7%, 1.3%, and 0%, respectively. The LOD for serous Hg was 0.1 μg/L, and no sample had a serous Hg level below the LOD.

### 3.2. The Association between Phthalate Esters and VBM

**Figure 1:**
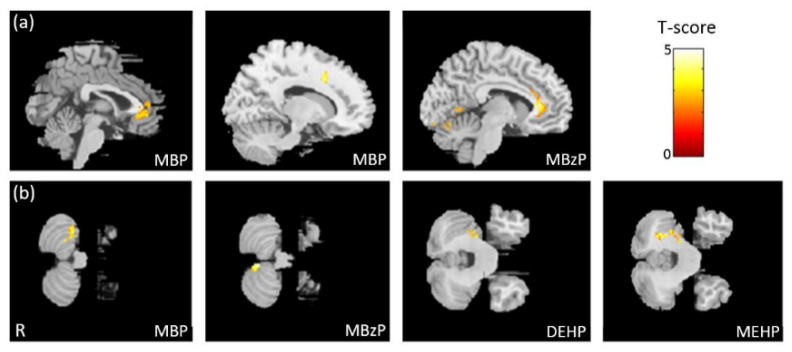
The association between concentrations of the phthalate esters and brain volume. We found (**a**) a negative association between MBP/MBzP concentrations and cingulate volume, and (**b**) a negative association between MBP/MBzP/DEHP/MEHP concentrations and cerebellum volume.

In the association between the maternal urine concentrations of phthalate esters and brain volume, which was corrected by using creatinine as a covariate, we found a negative association between MBP/MBzP concentrations and cingulate volume (Figure 1a), and a negative association between MBP/MBzP/DEHP/MEHP concentrations and cerebellum volume (Figure 1b). All of the above results were statistically significant (corrected *p* < 0.05). The color bar represents the T-score.

### 3.3. The Association between PFCs and VBM

**Figure 2:**
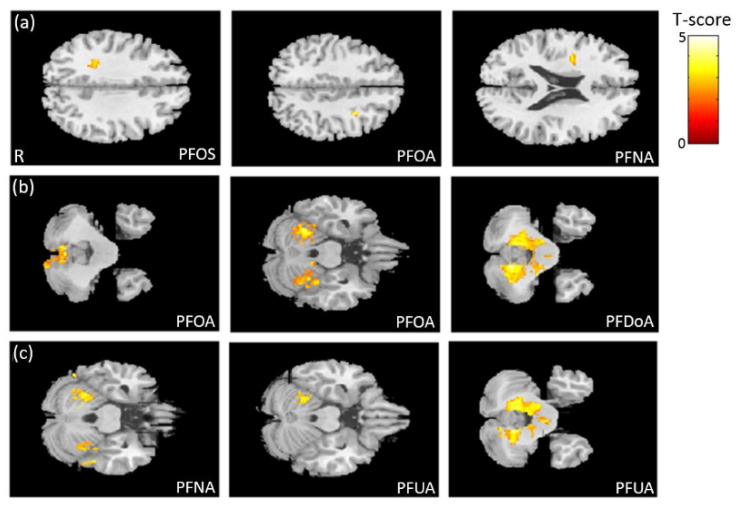
The association between concentrations of the PFCs and brain volume. We observed (**a**) a negative association between PFOS/PFOA/PFNA concentrations and frontal lobe volume, (**b**) a negative association between PFOA/PFDoA concentrations and cerebellum volume, and (**c**) a negative association between PFNA/PFUA concentrations and cerebellum volume.

In the association between the maternal blood concentrations of PFCs (PFOS, PFOA, PFDoA, PFNA, and PFUA) and brain volume, we observed a negative association between PFOS/PFOA/PFNA concentrations and frontal lobe volume (Figure 2a), a negative association between PFOA/PFDoA concentrations and cerebellum volume (Figure 2b), and a negative association between PFNA/PFUA concentrations and cerebellum volume (Figure 2c). All of the above results were statistically significant (corrected *p* < 0.05). The color bar represents the T-score.

### 3.4. The Association between Heavy Metals and VBM

**Figure 3:**
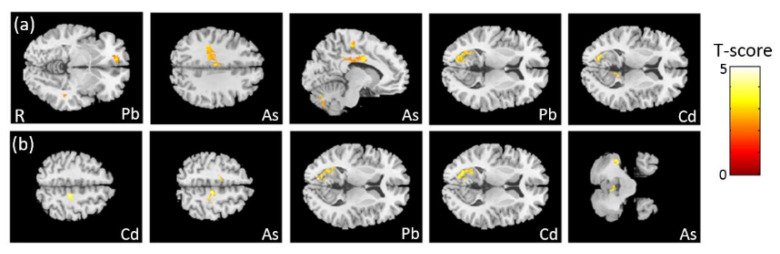
The association between concentrations of the heavy metals and brain volume, corrected by dividing by creatinine. We found (**a**) negative associations between Pb/As concentrations and frontal lobe volume, between As concentration and cingulate volume, and between Pb/Cd concentrations and left calcarine volume. In the association between concentrations of the heavy metals and brain volume, corrected by using creatinine as a covariate, we found (**b**) negative associations between Cd/As concentrations and frontal lobe volume, between Pb/Cd concentrations and left calcarine volume, and between As concentration and cerebellum volume.

In the association between the maternal urine concentrations of heavy metals (Pb, Cd, and As) and brain volume, which was corrected by dividing by creatinine concentration, we found negative associations between Pb/As concentrations and frontal lobe volume, between As concentration and cingulate volume, and between Pb/Cd concentrations and calcarine volume (Figure 3a). In the association between the maternal urine concentrations of heavy metals (Pb, Cd, and As) and brain volume, which was corrected by using creatinine as a covariate, we observed a negative association between Cd/As concentrations and frontal lobe volume, between Pb/Cd concentrations and calcarine volume, and between As concentration and cerebellum volume (Figure 3b). All of the above results were statistically significant (corrected *p* < 0.05). The color bar represents the T-score.

**Figure 4:**
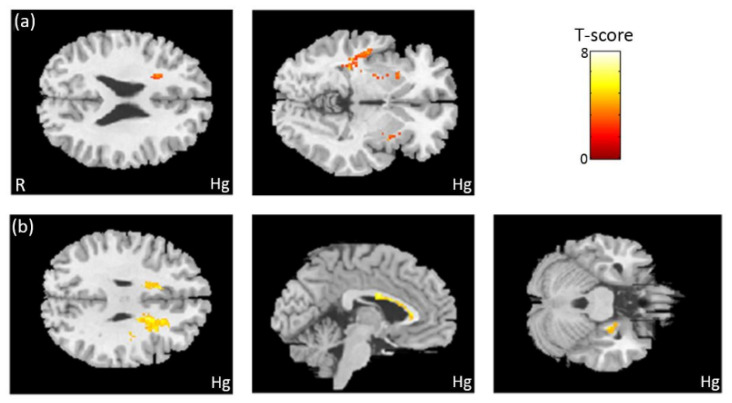
The association between concentration of Hg and brain volume. We observed (**a**) a negative association between Hg concentration in maternal serum and the volumes of the frontal and temporal lobes. We also found (**b**) a negative association between Hg concentration in umbilical cord blood and the volumes of the frontal lobe, corpus callosum, and right hippocampus.

In the association between the maternal blood concentration of Hg and brain volume, we observed a negative association between Hg concentration in maternal serum and the volume of the frontal and temporal lobes (Figure 4a). We also found a negative association between Hg concentration in umbilical cord blood and the volumes of the frontal lobe, corpus callosum, and hippocampus (Figure 4b). All of the above results were statistically significant (corrected *p* < 0.05). The color bar represents the T-score.

### 3.5. The Association between Phthalate Esters and GQI

**Figure 5:**
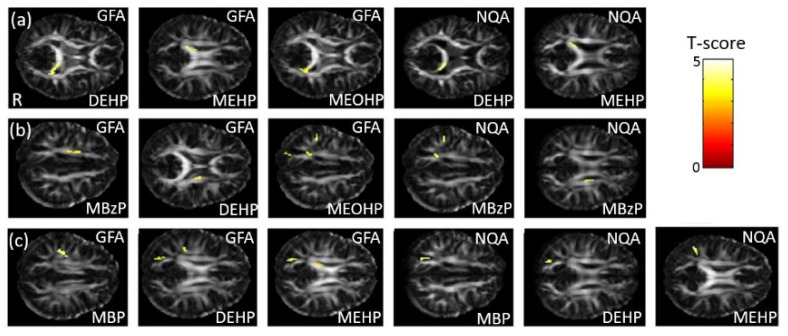
The association between concentrations of the phthalate esters and GQI. We found (**a**) a negative association between DEHP/MEHP/MEOHP concentrations and GFA, and between DEHP/MEHP concentrations and NQA, in the corpus callosum; (**b**) a negative association between MBzP/DEHP/MEOHP concentrations and GFA, and between MBzP concentrations and NQA, in the corona radiata; and (**c**) a negative association between MBP/DEHP/MEHP concentrations and GFA/NQA in the SLF.

In the association between the maternal urine concentrations of phthalate esters and GQI, which was corrected by using creatinine as a covariate, a significant negative association between DEHP/MEHP/MEOHP concentrations and GFA, and between DEHP/MEHP concentrations and NQA, in the corpus callosum (*p* < 0.005), were observed (Figure 5a). A significant negative association between MBzP/DEHP/MEOHP concentrations and GFA, and between MBzP concentrations and NQA, in the corona radiata (*p* < 0.004), were also found (Figure 5b). Moreover, we observed a significant negative association between MBP/DEHP/MEHP concentrations and GFA/NQA in the superior longitudinal fasciculus (SLF) (*p* < 0.007) (Figure 5c). The color bar represents the T-score.

### 3.6. The Association between PFCs and GQI

**Figure 6:**
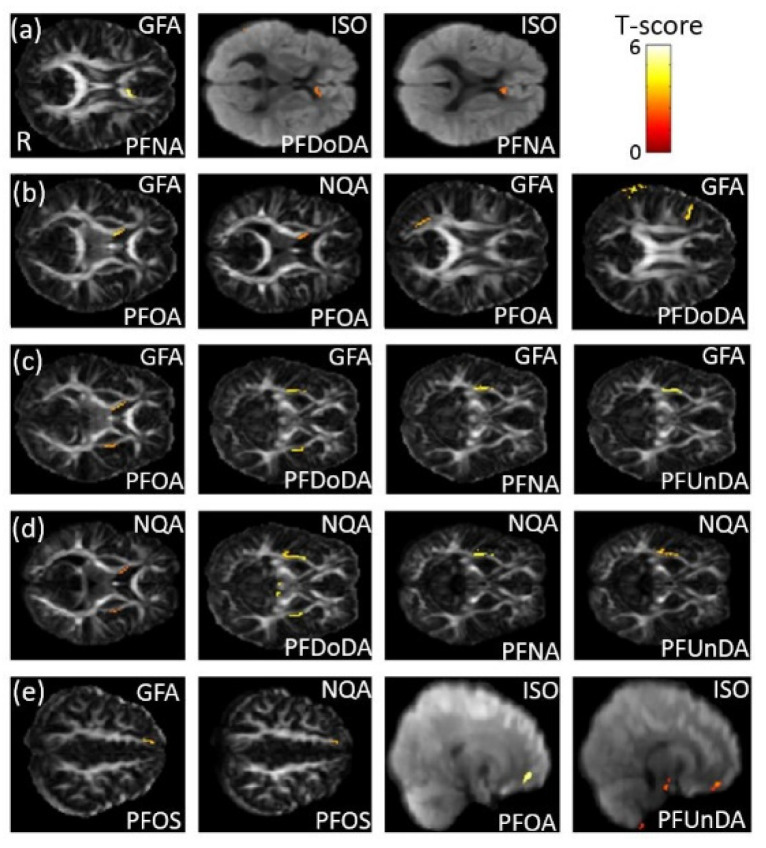
The association between concentrations of the PFCs and GQI. We found (**a**) a negative association between PFNA concentration and GFA, and a positive association between PFDoDA/PFNA concentrations and ISO, in the corpus callosum; (**b**) a negative association between PFOA concentration and GFA/NQA in the internal capsule, and a negative association between PFOA/PFDoDA concentrations and GFA in the SLF; (**c**,**d**) a negative association between PFOA/PFDoDA/PFNA/PFUnDA concentrations and GFA/NQA in the external capsule; and (**e**) a negative association between PFOS concentration and GFA/NQA, and a positive association between PFOA/PFUnDA concentrations and ISO, in the superior frontal gyrus (part of the SFO).

In the association between the maternal blood concentrations of perfluorides (PFOS, PFOA, PFDoA, PFNA, and PFUA) and GQI, a significant negative association between PFNA concentration and GFA, and a significant positive association between PFDoDA/PFNA concentrations and ISO, in the corpus callosum (*p* < 0.02), were observed (Figure 6a). A significant negative association between PFOA concentration and GFA/NQA in the internal capsule (*p* < 0.006), and a significant negative association between PFOA/PFDoDA concentrations and GFA in the SLF (*p* < 0.006), were also found (Figure 6b). A significant negative association between PFOA/PFDoDA/PFNA/PFUnDA concentrations and GFA/NQA in the external capsule (*p* < 0.02) was observed (Figure 6c,d). Moreover, a significant negative association between PFOS concentration and GFA/NQA, and a significant positive association between PFOA/PFUnDA concentrations and ISO, in the superior frontal gyrus—part of the superior fronto-occipital fasciculus (SFO) (*p* < 0.02)—were also found (Figure 6e). The color bar represents the T-score.

### 3.7. The Association Between Heavy Metals and GQI

**Figure 7:**
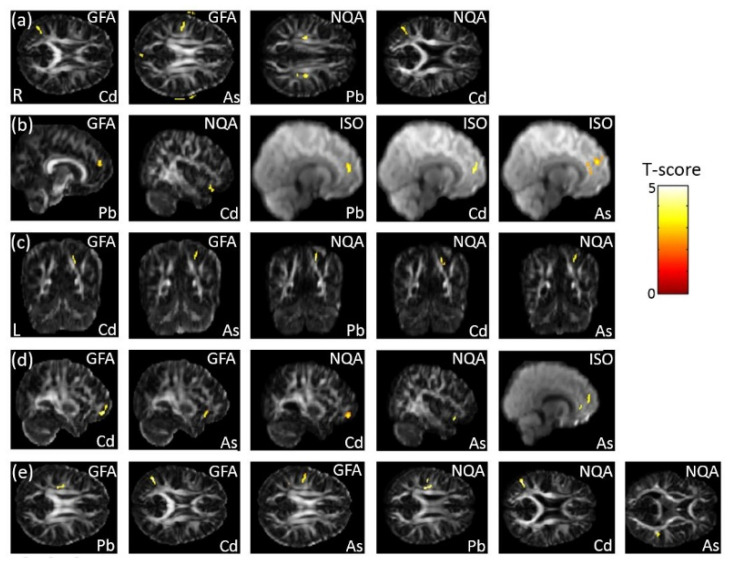
The association between concentrations of the heavy metals (Pb, Cd, and As) and GQI, corrected by dividing by creatinine. We found (**a**) negative associations between Cd/As concentrations and GFA, and between Pb/Cd concentrations and NQA, in the SLF; (**b**) negative associations between Pb concentration and GFA, and between Cd concentration and NQA, as well as a positive association between Pb/Cd/As concentrations and ISO, in the superior frontal gyrus (part of the SFO); and (**c**) a negative association between Cd/As concentrations and GFA, and between Pb/Cd/As concentrations and NQAn in the superior parietal gyrus (part of the SFO). In the association between heavy metal (Pb, Cd, and As) concentrations and GQI, corrected by using creatinine as a covariate, we found (**d**) a negative association between Cd/As concentrations and GFA/NQA, and a positive association between As concentrations and ISO, in the superior frontal gyrus (part of the SFO); while (**e**) a negative association between Pb/Cd/As concentrations and GFA/NQA in the SLF was also observed.

In the association between the maternal urine concentrations of heavy metals (Pb, Cd, and As) and GQI, which was corrected by dividing by creatinine concentration, we observed a significant negative association between Cd/As concentrations and GFA, and between Pb/Cd concentrations and NQA, in the SLF (*p* < 0.006) (Figure 7a). Significant negative associations between Pb concentration and GFA, and between Cd concentration and NQA, as well as positive association between Pb/Cd/As concentrations and ISO, in the superior frontal gyrus (part of SFO) (*p* < 0.02), were found (Figure 7b). In addition, a significant negative association between Cd/As concentrations and GFA, and between Pb/Cd/As concentrations and NQA, in the superior parietal gyrus (part of SFO) (*p* < 0.006), was also observed (Figure 7c). In the association between the urine concentrations of heavy metals (Pb, Cd, and As) and GQI, which was corrected by using creatinine as a covariate, a significant negative association between Cd/As concentrations and GFA/NQA, and a significant positive association between As concentration and ISO in the superior frontal gyrus (part of the SFO) (*p* < 0.008), were found (Figure 7d). A significant negative association between Pb/Cd/As concentrations and GFA/NQA in the SLF (*p* < 0.007) was also observed (Figure 7e). The color bar represents the T-score.

**Figure 8:**
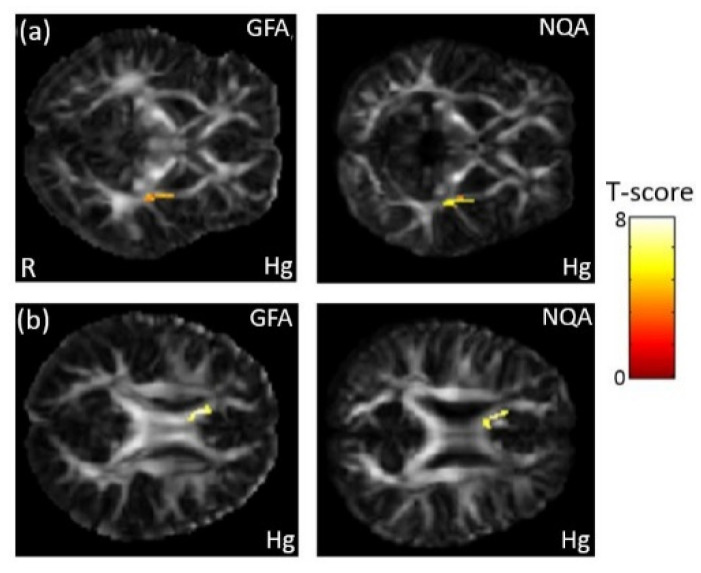
The association between concentration of the Hg and GQI. We found (**a**) a negative association between maternal serum Hg concentration and GFA/NQA in the external capsule, and (**b**) a negative association between umbilical cord blood Hg concentration and GFA/NQA in the corpus callosum.

In the association between the maternal blood concentration of Hg and GQI, a significant negative association between Hg concentration in maternal serum and GFA/NQA in the external capsule (*p* < 0.008) was observed (Figure 8a). A significant negative association between Hg concentration in umbilical cord blood and GFA/NQA in the corpus callosum (*p* < 0.005) was also found (Figure 8b). The color bar represents the T-score.

### 3.8. Pearson Partial Correlation between EDCs and GQI

The results of Pearson’s partial correlation showed significant moderate negative correlation between urine phthalate esters (creatinine as covariate) and GQI, including MBP and GFA in the SLF (*r* = −0.403, *p* = 0.008), MEOHP and GFA in the corona radiata (*r* = −0.350, *p* = 0.025), and DEHP and GFA in the corona radiata (*r* = −0.316, *p* = 0.044) and in the SLF (*r* = −0.372, *p* = 0.017); trends of moderate negative and positive correlation between blood PFCs and GQI, including PFUA and ISO in the external capsule (*r* = 0.308, *p* = 0.087) and in the SFO (*r* = 0.318, *p* = 0.076), and PFOA and GFA in the internal capsule (*r* = −0.350, *p* = 0.058); trends of significant mild to moderate negative correlation between urine heavy metals (corrected by dividing creatinine) and GQI, including Pb and GFA in the SFO (*r* = −0.305, *p* = 0.053) and NQA in the SFO (*r* = −0.299, *p* = 0.058), Cd and GFA in the SFO (*r* = −0.310, *p* = 0.048), and As and GFA in the SFO (*r* = −0.396, *p* = 0.020); significant moderate negative correlation between urine heavy metals (using creatinine as a covariate) and GQI, including Cd and GFA in the SFO (*r* = −0.323, *p* = 0.042), and As and GFA in the SFO (*r* = −0.372, *p* = 0.033); trends to significant moderate negative correlation between blood Hg (in maternal serum) and GQI, including GFA in the external capsule (*r* = −0.440, *p* = 0.078) and NQA in the external capsule (*r* = −0.496, *p* = 0.043), and blood Hg (in umbilical cord) and NQA in the corpus callosum (*r* = −0.334, *p* = 0.038) (Table 2 and Supplementary Table S1).

## 4. Discussion

### 4.1. Brain Volume and White Matter Structure Associated with Phthalate Esters

In the phthalate ester association analyses, we found that the higher the maternal urine concentrations of phthalate esters were, the more reduced the teenagers’ brain volumes, and this was mainly observed in the frontal lobe (cingulate) and the cerebellum. We also found that altered white matter integrity had a negative association with GFA/NQA, and a positive association with ISO, which were most evident in the corpus callosum, corona radiata, and SLF.

Several studies have pointed out that exposure to phthalate esters may induce abnormal frontal function and decrease IQ, which may degrade learning and cognitive abilities [5,6]. In addition, several cross-sectional studies have indicated that exposure to phthalate esters may be associated with attention-deficit/hyperactivity disorder (ADHD) symptoms, including inattention, aggression, and other emotional or behavioral problems in children [2,3]. Using MRI, several studies have revealed that ADHD was associated with cortical thickness in the frontal and anterior cingulate cortex [25,26]. In addition, a recent study found that the urine DEHP concentrations in children with ADHD were higher than those in children without ADHD, and the cortical thickness of the frontal lobes in children with ADHD was thinner than in children without ADHD [4]. Furthermore, several studies have shown that exposure to DEHP during pregnancy and lactation was associated with cerebellar-related emotional, cognitive, and social behavioral abnormalities [27,28,29]. A recent animal study revealed that maternal exposure to DEHP, and its metabolite MEHP, induced apoptosis of cerebellar granule cells, and the authors suggested that proliferation, differentiation, and apoptosis are pivotal steps during early postnatal cerebellar development, and that disturbances in any of these may lead to changes in cerebellar function and structure [30]. A recent systematic review and meta-analysis suggested that ADHD patients showed widespread abnormalities in brain white matter integrity, and that the affected white matter was consistently associated with fronto-striatal-cerebellar deficits [31].

### 4.2. Brain Volume and White Matter Structure Associated with PFCs

In the PFC association analyses, we found that the higher the maternal blood concentrations of PFCs were, the more reduced the teenagers’ brain volumes, and this was observed mainly in the frontal lobe and the cerebellum. We also found that measures of altered white matter integrity had a negative association with GFA/NQA and a positive association with ISO, and this was most evident in the corpus callosum, external and internal capsules, corona radiata, SFO, and SLF.

Previous animal studies have revealed that PFCs are able to cross the BBB, and can cause disruption to the central nervous system [32,33,34]. One animal study found that the cerebellum was susceptible to sodium fluoride, leading to neurodegenerative diseases [35]. Another animal study pointed out that PFCs can be distributed in the brain and cause cognitive deficits, and the authors also found that the decreases in the object recognition test were dependent on the PFC doses and concentrations in the brain [36]. In a human study using cross-sectional data, increased likelihood of ADHD in children was consistently associated with higher serum PFC levels [37]. Another recent study mentioned that prenatal exposure to PFNA was associated with ADHD symptoms among Asian children [38]. Furthermore, some studies have suggested that prenatal exposure to PFCs can affect motor function and result in malformation of the cerebellum, and that this exposure may even increase the risk of congenital cerebral palsy, abnormal behavior, mental retardation, and emotional disorder [12,39].

### 4.3. Brain Volume and White Matter Structure Associated with Heavy Metals

In the heavy metal association analyses, we found that the higher the maternal urine and blood concentrations of the heavy metals, the more reduced the teenagers’ brain volumes, and this was mainly observed in the frontal lobe, temporal lobe, hippocampus, cingulate, calcarine, corpus callosum, and cerebellum. We also found that altered white matter integrity had a negative association with GFA/NQA and a positive association with ISO that were most evident in the corpus callosum, external capsule, SFO, and SLF.

A review study suggested that Pb may induce brain alterations, mainly in the frontal lobe, hippocampus, cerebellum, and white matter myelination, which can lead to a variety of neurological disorders, such as brain damage, intellectual disability, and behavioral problems [40]. Some case reports have revealed that patients with inorganic Hg toxicity showed mild cortical atrophy and T2 hyperintensities in the frontal lobe and subcortical white matter [41,42]. Some review studies have shown that children exposed to Hg in the prenatal period had defects in attention, memory, language, and motor function, while workers exposed to Hg vapor showed induced motor and sensory neurotoxicity [43,44]. A cross-sectional study described neurobehavioral effects related to chronic As exposure in adolescents, and suggested that childhood exposure to As might affect neurobehavioral development in later life [45]. An animal study showed that concurrent exposure to Pb and As caused alterations in the hippocampus, midbrain, and cerebral cortex [46]. Another animal study showed that perinatal exposure to both Pb and Cd provoked neurochemical alterations in the cerebellum and the striatum [47].

Taken together, based on our study results, which are consistent with previous studies, we suggest that there are several specific vulnerable brain areas/structures associated with prenatal exposure to EDCs, including focal brain volume decreases—primarily in the frontal lobe, high frontoparietal lobe, temporooccipital lobe, and cerebellum—and white matter structural alterations, primarily in the corpus callosum, external and internal capsules, corona radiata, SFO, and SLF. Using VBM and GQI MRI evaluations, we could detect brain alterations caused by EDC exposure even at a subclinical stage, which may contribute to developing earlier diagnostic tools and perhaps disease prevention of EDC-associated neuropsychiatric disorders. Furthermore, the results of Pearson’s partial correlation analysis showed major trends of significant moderate correlation between EDCs and GOI indices in several white matter structures. However, we found that the image-based association analysis showed more sensitivity and specificity in detecting the locations of structural alterations, due to its voxel-wise (voxel by voxel) analysis method, and we recommended using image-based association analysis in future studies.

The method of creatinine correction is an important issue for urinary analyte-associated studies, but remains up for debate. Conventionally, the most popular method of standardizing analyzed urinary analyte concentrations is by dividing by the concentration of urinary creatinine. However, creatinine concentration could vary by gender, ethnicity, age, fat-free mass, and body mass index. Other experts recommend using creatinine as a covariate instead, and this approach allows the urinary analyte concentration to be appropriately adjusted for urinary creatinine, as well as allowing the statistical significance of other variables in the regression model to be independent of the effects of creatinine concentration [48]. Therefore, in our urinary EDC analysis, we mostly used creatinine as a covariate. Additionally, in urinary heavy metals analysis, we tried to use the two different creatinine correction methods for creatinine adjustment, and the results showed mostly consistent—but some varied—significantly associated brain structures (Figure 3 and Figure 7 and Table 2). Therefore, we cannot conclusively state the benefits of using creatinine as a covariate in our image-based regression study. Further study designs which focus on different creatinine correction methods are to be recommended.

There are several limitations to the present study that need to be considered. First, the number of recruited mother–child pairs was relatively small, which may limit the conclusions. Second, although we had accounted for certain possible confounders in our analyses, there are likely to be others that might have the effect of masking associations, and should be further explored in future studies. Third, the time interval between the prenatal exposure and the examination age in this study represents a time during which the teenagers may have experienced unknown levels of further exposure to EDCs during childhood, which was not accounted for, and so further longitudinal study is recommended. However, recent studies comparing both maternal EDC concentration and children’s EDC concentration with children’s behavior and brain structural changes showed a significantly greater impact of prenatal EDC exposure than that of childhood EDC exposure [3,49]. Finally, our study only recruited teenagers who had no neurologic or psychiatric disorders, which restricted us from being able to figure out clinical impact of the brain structural changes associated with prenatal EDC exposure. Further studies enrolling both normal participants and participants with neuropsychiatric or behavioral disorders may help to delineate a clearer picture of the cause–effect relationship. 

## 5. Conclusions

Using VBM and GQI MRI, we evaluated the association between prenatal exposure to EDCs—including phthalate esters, PFCs, and heavy metals (Pb, As, Cd, and Hg)—and subsequent brain structure changes. We found several specific vulnerable brain areas/structures, and these findings suggested that prenatal EDC exposure may play an important role in the development of future neuropsychiatric disorders. This study could draw attention to the issue of EDC exposure, and may inspire people to develop solutions for reducing EDC exposure throughout our lives.

## Figures and Tables

**Table 1:** EDC concentrations in urine/serum samples from pregnant women.

EDCs	*n*	Median	IQR
Urine (μg/g creatinine)			
As	45	35.89	24.05–45.69
Cd	47	0.79	0.52–1.12
Pb	47	3.51	2.31–4.13
MBP	49	69.54	37.71–144.71
MBzP	49	16.46	8.36–22.73
MEHP	49	17.21	8.08–30.74
MEOHP	49	15.76	7.50–38.76
DEHP	49	166.10 (mmole/g creatinine)	90.31–328.73
Serum (ng/mL)			
PFOA	47	2.51	1.20–3.13
PFOS	47	11.75	9.80–18.25
PFNA	47	1.24	0.59–1.81
PFUA	47	2.45	1.28–5.40
PFDoA	47	0.31	0.15–0.41
MeHg	21	6.50 (ug/L)	4.00–10.95

Note. DEHP = the molar sum of concentrations of three metabolites (MEHP, MEHHP, and MEOHP). IQR = interquartile range.

**Table 2:** Pearson’s partial correlation analysis between EDCs and GOI indices.

EDCs	Location_GQI Indices	*r*	*p*-Value
Urine phthalate esters			
MBP	SLF GFA	−0.403	0.008
MEOHP	CR_GFA	−0.350	0.025
DEHP	CR_GFA	−0.316	0.044
DEHP	SLF GFA	−0.372	0.017
Blood PFCs			
PFUA	EC_ISO	0.308	0.087
PFUA	SFO_ISO	0.318	0.076
PFOA	IC GFA	−0.350	0.058
Urine heavy metals (dividing creatinine)			
Pb	SFO GFA	−0.305	0.053
Pb	SFO_NQA	−0.299	0.058
Cd	SFO_GFA	−0.310	0.048
As	SFO_GFA	−0.396	0.020
Urine heavy metals (creatinine as covariate)			
Cd	SFO_GFA	−0.323	0.042
As	SFO_GFA	−0.372	0.033
Blood Hg			
maternal Hg	EC_GFA	−0.440	0.078
maternal Hg	EC_NQA	−0.496	0.043
umbilical cord Hg	CC_NQA	−0.334	0.038

Note 1: Gender, IQ, and family income were used as covariates, and only the results of *p* < 0.1 are shown in the table. Note 2: SLF = superior longitudinal fasciculus; CR = corona radiata; EC = external capsule; IC = internal capsule; SFO = superior fronto-occipital fasciculus; CC = corpus callosum.

## Data Availability

The datasets generated during and/or analyzed during the current study are available from the corresponding author on reasonable request.

## Supplementary Materials

The following are available online at https://www.mdpi.com/article/10.3390/ijerph18094798/s1, Table S1: Pearson partial correlation analysis between EDCs and GOI indices.
