# Supplementary material for: Prenatal Exposure to Endocrine-Disrupting Chemicals and Subsequent Brain Structure Changes Revealed by Voxel-Based Morphometry and Generalized Q-Sampling MRI
Source: Int J Environ Res Public Health. 2021 Apr 30;18(9):4798. doi: 10.3390/ijerph18094798 (PMC8125311; doi:10.3390/ijerph18094798)
Supplement: Supplementary file 1 [file ijerph-18-04798-s001.zip › ijerph-1149568-supplementary.pdf]

Table S1. Pearson partial correlation analysis between EDCs and GOI indices

EDCs		Location_GQI indices								
**Urine phthalate esters**		**CC_GFA**	**CC_NQA**	**CC_ISO**	**SLF_GFA**	**SLF_NQA**	**SLF_ISO**	**CR_GFA**	**CR_NQA**	**CR_ISO**
**MBP**	**r**	-0.220	-0.123	0.137	-0.403	-0.103	0.172	-0.108	0.031	0.150
	**p-value**	0.161	0.438	0.386	0.008*	0.516	0.276	0.495	0.847	0.343
**MBzP**	**r**	-0.233	-0.257	-0.204	-0.228	-0.197	-0.146	-0.041	-0.073	-0.148
	**p-value**	0.148	0.110	0.207	0.157	0.223	0.368	0.803	0.654	0.361
**MEHP**	**r**	-0.086	0.052	-0.102	0.018	0.128	-0.162	-0.108	0.055	-0.137
	**p-value**	0.589	0.743	0.521	0.910	0.419	0.305	0.495	0.730	0.387
**MEOHP**	**r**	-0.129	-0.010	-0.114	-0.234	-0.029	-0.049	-0.350	-0.097	-0.045
	**p-value**	0.421	0.950	0.477	0.140	0.857	0.760	0.025*	0.548	0.778
**DEHP**	**r**	-0.239	-0.040	-0.117	-0.372	-0.051	-0.107	-0.316	-0.068	-0.093
	**p-value**	0.133	0.804	0.468	0.017*	0.750	0.506	0.044*	0.672	0.565
**Blood PFCs**		**EC_GFA**	**EC_NQA**	**EC_ISO**	**SFO_GFA**	**SFO_NQA**	**SFO_ISO**	**IC_GFA**	**IC_NQA**	**IC_ISO**
**PFOS**	**r**	-0.162	-0.049	0.104	-0.175	-0.260	0.026	-0.084	-0.056	0.110
	**p-value**	0.320	0.763	0.523	0.279	0.105	0.872	0.601	0.714	0.501
**PFUA**	**r**	-0.182	-0.020	0.308	-0.079	0.088	0.318	-0.130	0.040	0.259
	**p-value**	0.318	0.916	0.087**	0.668	0.634	0.076**	0.479	0.827	0.153
**PFOA**	**r**	-0.150	-0.073	0.128	-0.273	-0.166	0.085	-0.350	-0.114	0.017
	**p-value**	0.429	0.703	0.500	0.145	0.382	0.654	0.058**	0.550	0.930
**PFNA**	**r**	-0.215	0.000	0.093	-0.130	-0.063	0.059	-0.164	0.077	0.080
	**p-value**	0.188	0.998	0.575	0.430	0.703	0.722	0.320	0.640	0.627
**PFDoA**	**r**	-0.247	-0.016	0.105	-0.119	0.021	0.101	-0.138	0.106	0.097
	**p-value**	0.188	0.935	0.579	0.530	0.913	0.594	0.466	0.576	0.610

Urine heavy metals (dividing creatinine)		SFO_GFA	SFO_NQA	SFO_ISO	SLF_GFA	SLF_NQA	SLF_ISO
Pb	r	-0.305	-0.299	-0.106	-0.167	-0.138	-0.016
	p-value	0.053**	0.058**	0.508	0.298	0.389	0.919
Cd	r	-0.310	-0.164	-0.110	-0.156	0.109	0.034
	p-value	0.048*	0.307	0.492	0.331	0.498	0.832
As	r	-0.396	-0.245	0.113	-0.267	-0.015	0.207
	p-value	0.020*	0.162	0.525	0.128	0.933	0.241
**Urine heavy metals** (creatinine as covariate)							
Pb	r	-0.245	-0.260	-0.119	-0.173	-0.193	0.003
	p-value	0.128	0.105	0.463	0.285	0.232	0.984
Cd	r	-0.323	-0.149	-0.126	-0.164	0.062	0.059
	p-value	0.042*	0.358	0.439	0.313	0.703	0.718
As	r	-0.372	-0.258	0.091	-0.247	-0.103	0.214
	p-value	0.033*	0.147	0.616	0.165	0.568	0.231

Blood Hg		EC_GFA	EC_NQA
maternal Hg	r	-0.44	-0.496
	p-value	0.078**	0.043*
umbilical cord Hg	r	-0.114	-0.334
	p-value	0.489	0.038*

Note 1: Gender, IQ and family income were used as covariates.

Note 2: * p<0.05, ** p<0.1

Note 3: SLF= superior longitudinal fasciculus, CR= corona radiata, EC= external capsule, IC= internal capsule, SFO= superior fronto-occipital fasciculus, CC= corpus callosum.
